# Diagnosis and management of the infected total knee replacement: a practical surgical guide

**DOI:** 10.1186/s40634-021-00333-2

**Published:** 2021-02-22

**Authors:** Akos Zahar, Martin Sarungi

**Affiliations:** 1 Department of Orthopaedic & Trauma Surgery, Helios Klinikum Emil von Behring, Walterhöferstrasse 11, 14165 Berlin, Germany; 2 Orthopaedic Department, The Golden Jubilee National Hospital, Glasgow, Scotland, UK

**Keywords:** Prosthetic joint infection (PJI), Total knee arthroplasty (TKA), Diagnostic protocol, Surgical treatment, One-stage septic exchange, Two-stage revision

## Abstract

**Purpose:**

Prosthetic joint infection (PJI) after total knee arthroplasty (TKA) is a significant burden in health care. Diagnosis and proper management are challenging. A standardised procedure for the diagnostic workup and surgical management provides clear benefits in outcome.

**Methods:**

Several diagnostic protocols and definitions for PJI were established in recent years. Proper PJI diagnosis remains critical for success and for choosing the optimal treatment option. A distinct workup of diagnostic steps, the evaluation of the results in a multidisciplinary setup and the meticulous surgical management of the infection are the key factors of successful treatment.

**Results:**

The management of PJI after TKA consists of early revision with debridement and implant retention (DAIR) in early cases or staged revision in late infections beyond 30 days postoperative or after onset of acute symptoms. The revision is performed as a two-stage procedure with the use of a fixed or mobile antibiotic spacer, or in selected cases as a single-stage operation with the use of local and systemic antibiotic treatment.

**Conclusions:**

This paper reflects the opinion of two revision surgeons who follow the same protocol for diagnosis and treatment of PJI after TKA, highlighting the key steps in diagnosis and management.

**Level of evidence:**

Expert’s opinion

## Introduction

Prosthetic joint infection (PJI) remains a devastating complication after total knee arthroplasty (TKA), it is the most common cause of revision for failed knee replacement (16.8 to 25.2%) [3]. As a matter of fact, both diagnosis and treatment are challenging. Several protocols were introduced in recent years providing widely used algorithms for decision-making. Novel diagnostic tools and scores make it possible to diagnose PJI much easier than a decade ago. There are several clearly defined treatment options after PJI, depending on the virulence of the causative agent and the time of onset of the symptoms. Treatment may involve debridement, antibiotic therapy and implant retention (DAIR), and exchange arthroplasty with implant removal as two-stage procedure or in certain cases as a single-stage revision.

The clinical suspicion of PJI should always be considered when a TKA becomes painful, since pain is still an important factor. Especially during 0–24 months after primary arthroplasty, when the painful joint indicates an early failure, diagnostic workup of the TKA is mandatory. Local signs of infection may support the clinical suspicion, but pain is the leading symptom in > 90% of the cases. In the presence of a draining sinus, the TKA is always considered as infected and staged revision should be scheduled.

Proper PJI diagnosis remains critical for success and choosing the optimal treatment option [7]. Recently, national and international workgroups have convened to establish standardized diagnostic protocols for suspected PJI. In 2010, the American Academy of Orthopaedic Surgeons Clinical Practice Guidelines on Diagnosis of Periprosthetic Joint Infection were published [6, 12]. Soon after, the Musculoskeletal Infection Society (MSIS) and the Infectious Disease Society of America (IDSA) devised criteria to standardize the definition of PJI in 2011 [14, 17]. The International Consensus Meeting (ICM) for PJI in 2013 then endorsed the MSIS definition and modified it slightly [15]. These definitions have now become widely established among orthopaedic surgeons worldwide and their use has significantly improved clinical decision-making, as well as diagnostic research, by allowing for consistency between studies and enhancing the potential for collaboration. Most recently, a new 2018 evidence-based PJI definition has been published which demonstrates improved performance for diagnosing hip and knee PJI on formal external validation [16]. The latest definition of PJI with a practical guide for clinicians, based on a three-level approach was released by the European Joint Infection Society (EBJIS) by the end of 2020 [10].

Which evidence-based diagnostic options are really useful and should be carried out? Which preoperative diagnostic protocols are recommended? Which surgical options can be emphasized after a workup in a multidisciplinary setup?

## Diagnostic options

The first line of preoperative diagnosis in suspected PJI is plain radiographs, blood tests and aspiration of the painful joint. The radiographs in two planes are checked for solid implant fixation (radiolucent lines), presence of osteolytic changes of distal femur/proximal tibia, periarticular ossifications. To date, there is no single clinical tool to predict PJI with a 100% accuracy. Therefore, a panel of different diagnostic methods should be applied for diagnosing PJI. Blood tests should focus on leukocyte count, differential, C-reactive protein (CRP) level and erythrocyte sedimentation rate (ESR).

CRP and ESR have variable sensitivities and specificities, as reported in the literature. In a recent study [2] the pooled data of 3909 patients revealed a sensitivity for ESR and CRP, of 75% and 88% respectively. That means, round 20% of the patients with a proven TKA may have a normal CRP or ESR. Beside CRP, the level of D-dimer can be analysed in the blood test as an alternative or in addition. Elevated D-dimer levels predict the likelihood of a PJI.

In acute haematogenous infections patients may have fever (> 38 °C), in these cases blood cultures may be obtained in order to detect bacteraemia at an early stage, before antibiotic treatment is introduced. These patients may have high levels of CRP (> 100 mg/L), pathologic levels of procalcitonin (PCT), visible purulence in the joint aspirate and a tendency towards systemic inflammatory response syndrome (SIRS) with multiorgan failure (elevated heart rate, low systolic pressure). Early surgery with irrigation and drainage of the knee is mandatory, in order to save the patient from septicaemia.

Aspiration of the painful knee under antiseptic conditions (in OR or similar clean environment, but not in the outpatient clinic) is the key diagnostic step in the work-up of PJI. Whenever possible, an antibiotic holiday (> 14 days) is achieved, in order to avoid false negative results [21]. The painful knee is prepped and an aspirate of joint fluid is obtained with sterile tools, including sterile surgical gloves and gowns. These measures are important in order to avoid contamination. A skin incision is normally not necessary. A panel of investigations can be carried out, that is why a minimum of 2 mL fluid is optimal and admixture of blood is to be avoided.

**Fig. 1:**
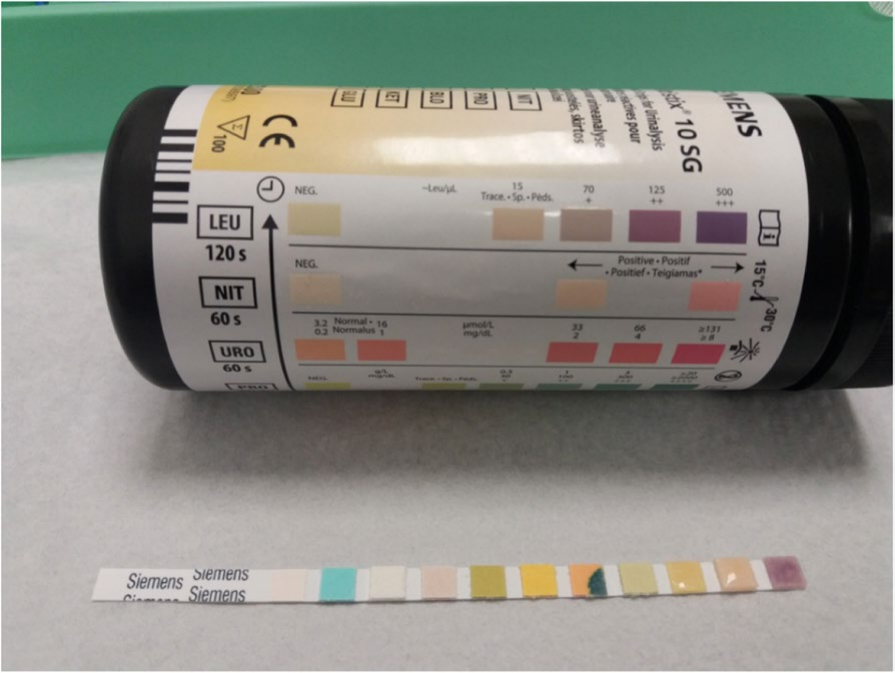
Positive leukocyte esterase test (LET): Please note the first test strip on the right hand side; it has turned purple after 2 min which equals LET +  +  + according to the scale on the box above the stripe

At the site of aspiration bedside tests can be performed. The leukocyte esterase dip stick test (LET) is a cheap tool allowing for quick decision making in both preoperative and intraoperative diagnostics. If the test is positive (deep purple colour, + +) PJI is most probably considered, but other conditions with inflammation (gout, pseudo gout, rheumatoid disease, etc.) should be ruled out [Fig. 1]. If the test is negative (white or light pink colour), PJI is very much unlikely. In a meta-analysis of evidence based studies with a total of 1.011 patients showed a pooled sensitivity of 90% (95% CI, 76 to 96%) and a specificity of 97% (95% CI, 95 to 98%) [22].

Another good but more expensive option as a point-of-care test is the Alpha Defensin lateral flow test with quite a high sensitivity and specificity, providing additional information about possible infection. Once the test is negative, PJI can be ruled out with a high certainty. A positive test can include other conditions, like polyethylene wear or metal debris. Nevertheless, it is a reliable tool in the panel of PJI diagnostics. The accuracy can be improved with the lab based ELISA version of the test, if available [8].

The synovial fluid should be sent to the lab, where admixture of blood can be eliminated by centrifugation. An analysis of the cell count (WBC) and the percentage of granulocytes (PMN%) is performed. This is a very valuable tool and should be available in every orthopaedic centre. The cut-off level of these parameters is subject to debate, but an elevated cell count (above 3000) and a high PMN% (> 80%) provide a sensitivity and specificity more than 90%. In a recent study, cutoff levels for PJI diagnosis after TKA were 1630 leukocytes/μL (SE 83.6%, SP 82.2%) and a PMN% of 60.5% (SE 80.3%, SP 77.1%) [25].

The gold standard of PJI diagnostics is still bacterial culture, which is carried out in the microbiology lab for at least 14 days. Two positive cultures of the same organism are enough for diagnosis, but in the most cases there is only one positive culture from the aspiration and the second positive sample is from the intraoperative biopsies.

**Fig. 2:**
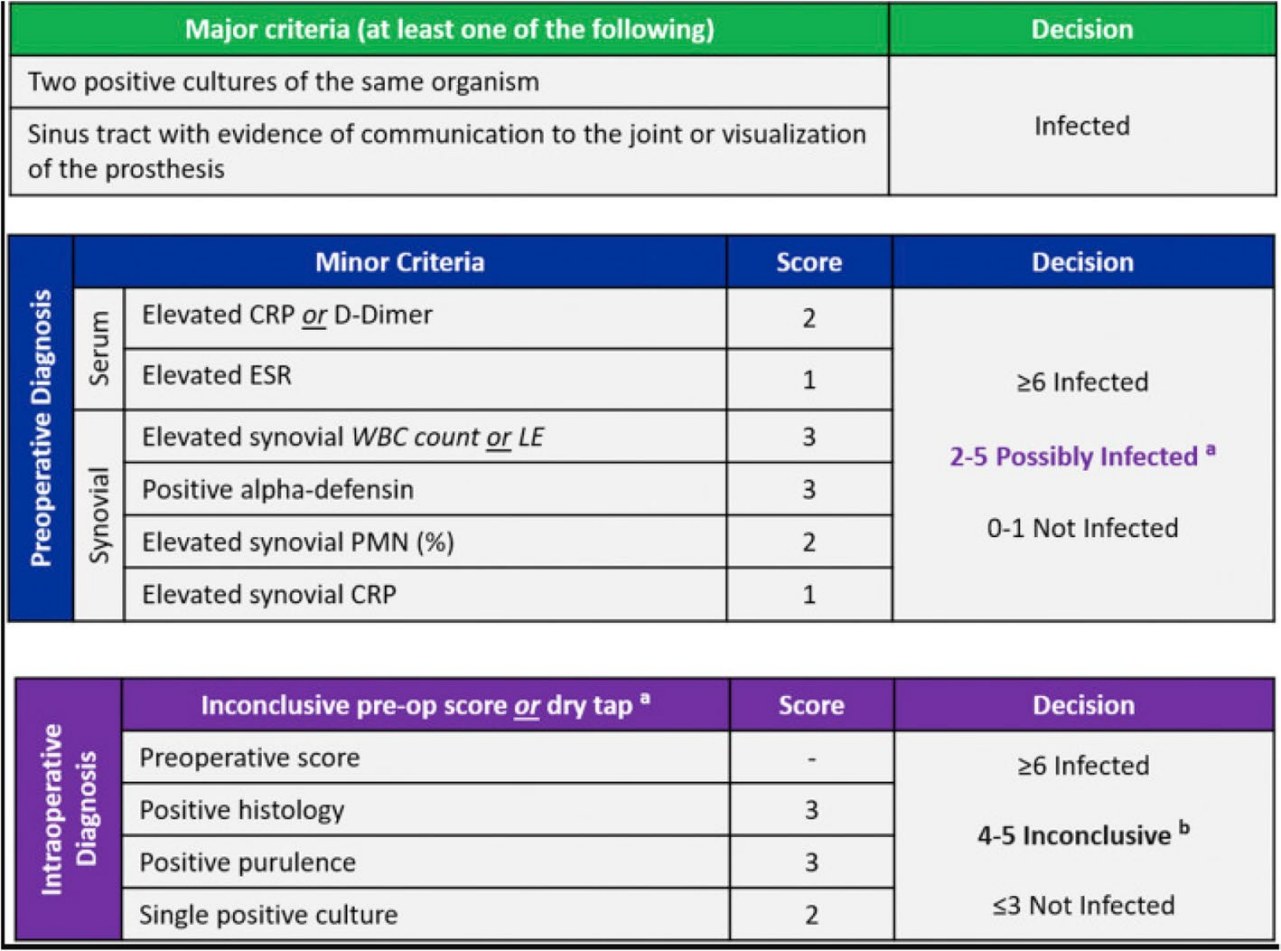
New scoring system based on 2018 ICM Philadelphia (courtesy of Journal of Arthroplasty) definition for PJI. Caution is required in several conditions, like adverse local tissue reaction, crystal deposition disease, slow growing organisms, etc. [16]

Do date, there is no single test that provides clear evidence of PJI, therefore we use the above mentioned series of investigations. The results of these tests are evaluated in a multidisciplinary team (MDT) setup and the diagnosis of PJI along with treatment options is defined [12]. A useful tool with reliable performance for diagnosing PJI after TKA is a scoring system based on the 2018 Philadelphia Definition of hip and knee infection [9] [Fig. 2]. Using this score, patients with an aggregate score of greater than or equal to 6 are considered having PJI, while a score 2 to 5 require further workup and evaluation of intraoperative samples. A score 0 or 1 means no evident infection.

## Treatment – general considerations

There are different options in the management of infected TKA [4], but regardless of the chosen treatment, the key general considerations for the local set-up must include:

- Early correct diagnosis of infection (as described above);
- Established multidisciplinary team (MDT) work including experienced surgeon(s), microbiologist(s) and/or infection disease doctor(s) and others;
- Regular MDT meetings to discuss and document the treatment plan for every case;
- Careful documentation of surgical outcomes and regular morbidity and mortality meetings to present results;
- Easy access to other vital services as required such as plastic surgery, vascular surgery, high dependency unit.

### Debridement antibiotic treatment and implant retention (DAIR)

#### Definition

Eradicate the prosthetic joint infection by performing a radical surgical debridement of infective tissue; a massive lavage of the joint with an antiseptic solution, using jet-lavage; changing all modular accessible part of the implants (polyethylene inlay); and administering high dose of targeted antibiotic treatment in the postoperative period.

#### Indication

1. the early postoperative PJI, within 4 weeks after surgery, before mature biofilm formation. 2. acute haematogenous PJI which presents itself with a short (maximum 4 weeks) onset with well-fixed implants and intact soft tissues.

#### Relative indication

Established late(> 4 weeks) PJI in the presence of megaprosthesis (e.g. total femur implant), where primary soft tissue closure is possible, implant is well fixed and the patient is not fit for full implant revision, but modular parts can be changed with ease.

#### Contraindication

1. Late chronic PJI with lose implant. 2. Infection with soft tissue defects that will make primary skin closure impossible. 3. Severely unwell patient not tolerating any surgical intervention.

#### Procedure

DAIR procedure should be viewed as a difficult “single stage revision” operation where parts of the implant are retained, and it is often the last chance to save the implant before full revision. The procedure should be carried out by experienced arthroplasty surgeon (preferably revision arthroplasty surgeon), all modular parts of the implant should be exchanged during the procedure, and wherever possible it should be done avoiding out-of-ours when generally less experienced staff available.

1. Aggressive radical soft tissue debridement and removal of all modular components together with any necrotic infected tissue, including the difficult-to-access areas such as the posterior capsule of the knee joint.
2. Several samples (5 samples recommended) should be taken, each with sterile set of instruments, and from specific standard location (e.g. synovium, under tibia polyethylene, posterior capsule, femoral and tibial implant membranes). All samples should be labelled clearly regarding their location and should also be reported the same way from microbiology.
3. Pulsatile low-pressure jet lavage (3–6 L), and antiseptic solution (e.g. povidon-iodine) are used. The surgical debridement with irrigations continued in cycles until satisfactory clean surgical field is achieved.
4. Mechanical cleaning of the retained visible femoral and tibial components (with sterile surgical plastic brushes, which do not damage the metal surfaces) are recommended.
5. The surgical team at this point change into new gloves, the surgical area is re-draped and a new, sterile set of surgical tray is used for the second part.
6. New sterile modular implants are inserted, the use of absorbable antibiotic loaded carrier (such as calcium sulphate beads) can increase the local antibiotic delivery, and the wound is closed in a multilayer fashion. A watertight closure is essential.
7. Surgical drain if be used generally removed after 48 h.
8. DAIR is always followed by antibiotic treatment (2 weeks iv., 4 to 10 weeks oral, depending on the causing organism and discussion with the microbiologist/infection diseases specialist in the MDT setup).

### Two-stage septic revision

#### Definition

Two surgical interventions, shorter (2–4 weeks) or longer (8–12 weeks) interval between them. During the first operation the implant is fully removed with all cement and foreign material, aggressive, extensive surgical debridement is performed to remove all infected, necrotic soft tissues and bone parts, and static or mobile antibiotic loaded spacer is used to allow the tissues to heal and the patient to mobilize. Once the infection is eradicated, during the second operation the spacer is removed and a new prosthesis is re-implanted. Targeted antibiotic treatment is carried out between the first and second stage operations, and after re-implantation.

#### Indication

1. Chronic late PJI, with loose implants, where the causing organism is not known (culture negative PJI). 2. PJI with difficult to treat (DTT) organism(s). 3. PJI with extensive soft tissue and/or bone defects, which would require additional extensive reconstruction. 4. Unhealthy host (McPherson Type B or C) [11]. 5. Remote source of infection in another localisation.

#### Relative indication

1. Late PJI where single stage revision procedure could be performed, but the local team has no expertise with that procedure. 2. Previous multiple failed revisions for infection.

#### Contraindication

1. Early postoperative infection (< 4 weeks) with well-fixed implants. 2. Patient unfit for surgery.

#### Procedure

Requires experienced revision surgical team and appropriate setup.

- 1 to 5. surgical steps are same as described in DAIR, but the implant and all cement are fully removed in every case, if necessary, osteotomies are utilized [1, 23].
- 6. Once clear surgical field is achieved, the surgical team change into new gown/gloves, the surgical area is re-draped and a new, sterile set of surgical tray is used.
- 7. Static or mobile spacers of antibiotic loaded acrylic bone cement (ALAC) are implanted and wound closed either primarily or with the help of plastic surgeon to address significant soft tissue defects at this stage. Extensive bone or soft tissue damage is addressed with a fixed spacer rather than with a mobile one, which is used in more stable knees with intact collateral ligaments. Antibiotic content in ALAC can exceed 10% volume for spacers (up to 20%).
- 8. In culture negative PJI intravenous broad spectrum antibiotic treatment is administered, otherwise targeted treatment is commenced immediately after surgery and it is reviewed once the microbiology results from intraoperative tissue samples are available.
- 9. Continue with antibiotic therapy (shorter 2–4 weeks or longer 6–12 weeks, depending on the causing organism and discussion with infection specialist). In the most cases, we wait 6 weeks after first stage procedure. Monitor CRP, wound, body temperature and overall clinical picture, on a regular basis.
- 10. Consider soft tissue reconstructions such as flaps as required in cases with massive skin defects. Consult the plastic surgeon about timing of a gastrocnemius or other musculocutaneous flap, when indicated. We recommend the use of negative pressure treatment (VAC) at first stage procedure to cover the defects. The VAC is then changed 2 to 3 times and when infection is under control musculocutaneous flap can be performed. At time of re-implantation the transplanted soft tissues must be solid in order to achieve a primary wound closure.
- 11. Once infection is settled (CRP at baseline, wound is healed, soft tissues are normal, knee is painless, body temperature is normal), second stage procedure is performed: repeat surgical steps 1 to 5 with removal of spacer. There is a second chance for aggressive debridement of the bone and soft tissues.
- 12. Re-implant the new prosthesis addressing the necessary bony or soft tissue defects at this time (bone reconstructions with metal sleeves, porous metal cones or allografts), using ALAC again for fixation. When using uncemented implants, consider other options for local antibiotic delivery, such as calcium sulphate beads. For adequate cement strength the antibiotic volume should not exceed 10% (premanufactured ALAC is preferable, rather than hand mixed).
- 13. Targeted post op antibiotic therapy as per recommendation from the infection specialist. Postoperatively monitor CRP, wound healing, general clinical condition. In case of suspected re-infection consider tests as described above.

#### The role of spacers

**Fig. 3:**
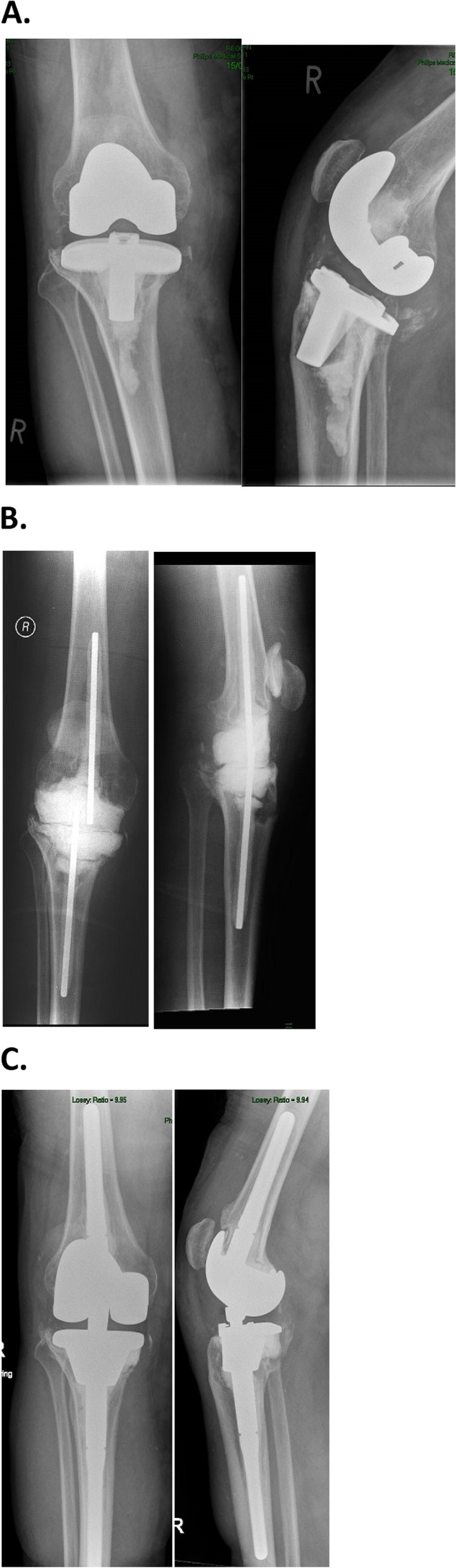
Radiographs (a-p and lateral view) of a surgical case, right knee TKA after low grade infection with coagulase-negative *Staphylococcus* 2 years post-op with catastrophic implant failure (**a**), after implant removal and 1st stage static antibiotic cement spacer armed with IM femoral and tibial rods (**b**), and 2nd stage revision with tibial metal cone and rotating hinge revision implant, 3 years post-op (**c**)

The static or mobile spacers serve a dual purpose:

1. **Very high dose of local antibiotic delivery**. Plan the appropriate use of antibiotic bone cement prior to surgery: either ready-made antibiotic bone cement, or manually mix the targeted antibiotics to the bone cement as per the recommendation of the infection specialist doctor. For spacer it is possible to use much higher dose of antibiotics to the cement than the recommended max 10% volume, because the fixation is only temporary.
2. **To allow the patient to mobilise.** between the first stage and second stage procedure. **Static spacer** is generally recommended with larger bone defects when mobile spacer would not be stable enough or in case of extensive soft tissue defects where plastic surgery is required during or after the first stage procedure. Static spacers should be inserted with mild distraction of the knee joint, to allow sufficient stability for the patient to mobilise with a brace and partial weight bearing after surgery. The stability of the static spacer could be enhanced with a pair of femoral and tibial intramedullary metal rods, which prevent spacer dislocation and allow intramedullary antibiotic delivery at the same time [Fig. 3]. Mobile spacers can be either ready made, with industry mixed antibiotic content, or bespoke using single-use moulds and individual mixed ALAC. They are more expensive than static spacers, but they have the additional benefit that the patient can have some degree of movement in the knee joint between the stages. This helps with potential quicker postoperative recover, but meta-analysis comparing the use of mobile and static spacers did not find significant difference regarding the final outcome.

### Single-stage revision

#### Definition

**Fig. 4.**
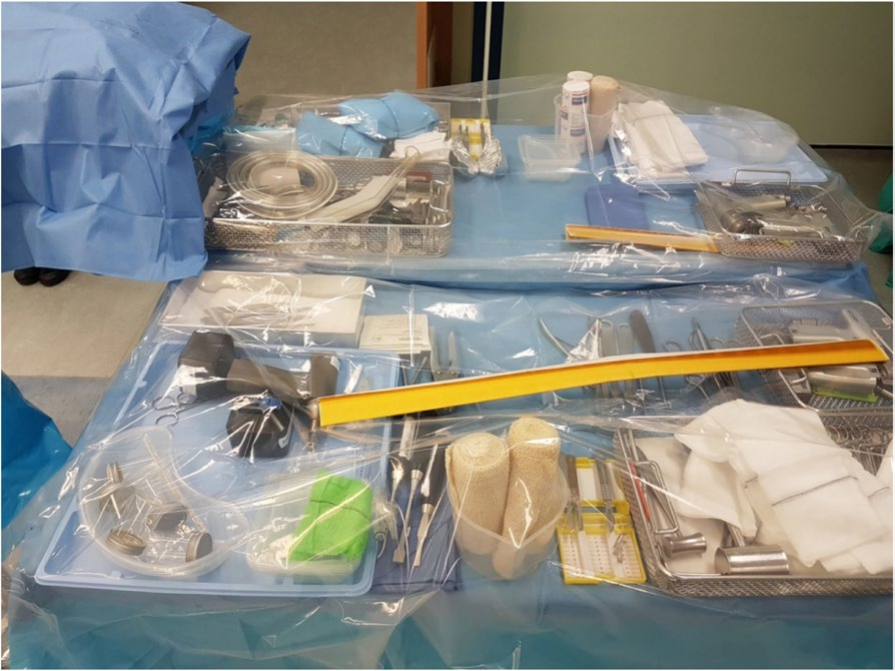
2-in-1 single stage revision basic orthopaedic tray setup, consists of two basic trays: 1st basic orthopaedic tray is for implant removal and the 2nd is for re-implantation (plus revision trays as necessary). Please note that the tray at the front is the implant removal tray (5 specimen pots, multiple instruments for intraoperatve samples)

Single-stage revision is aimed to fully remove the implant with all bone cement, perform an aggressive, extensive surgical debridement removing all infected, necrotic soft tissues and bone parts, and re-implant a new prosthesis during the same operation. Whilst it is one operation, it is usually done in two parts with the surgical team re-scrubbing, re-draping and using new sterile set of instruments for the second part of the surgery. Because of this, single-stage revisions for PJI are also called “2-in-1” revisions, requiring a special setup in the OR [Fig. 4].

#### Indication

1. Chronic late PJI, with loose or solid implants, where the causing organism is known. 2. Good host (McPherson type A)

#### Relative indication

PJI with difficult to treat (DTT) organism. Which germs are difficult to treat is a question of available antiinfective agents, resources. Methicillin-resistant *Staphylococcus aureus* (MRSA) and *E. faecalis* are considered to be DTT [13, 19]. Some revision centres perform single-stage revision even in these cases, but poorer outcomes may be expected [5].

#### Contraindication

1. Early postoperative infection (< 4 weeks) with well fixed implants. 2. Chronic late PJI with extensive soft tissue and/or bone defects which require extensive reconstruction and primary wound closure cannot be achieved. 3. Lack of experience with single-stage revision. 4. Poor host (McPherson type C). 5. Patient unfit for surgery (e.g. acute sepsis with multiorgan failure).

#### Procedure

Requires experienced surgical team with this type of procedure and appropriate local setup [24].

1. 1 to 6 surgical steps are same as described in two-stage procedure.
2. Re-implantation of the new prosthesis, addressing the necessary bone defects at this time; primary wound closure is essential.
3. Use uncemented fixation with other antibiotic delivery option or cemented fixation with ALAC. For adequate cement strength the antibiotic volume should not exceed 10% of the cement volume.
4. Targeted post operative antibiotic therapy as per recommendation from the infection specialist doctor in the MDT setup (e.g. short terms iv. followed by longer term oral therapy, duration 2 to 12 weeks) [20];
5. The postoperative care and monitoring is similar as described in step 13 of the two-stage revision.

## Discussion

It is widely accepted that the successful treatment of infected TKA depends on early correct diagnosis and well-coordinated management by experienced multidisciplinary team. The current paper focused on the practical surgical aspects of the most common surgical procedures to treat the infected TKA (DAIR, two-stage and single stage revision), but it is important to be aware of and familiar with other treatment options for selected cases, which are used less frequently (such as long term antibiotic suppression, knee fusion, above knee amputation).

Whilst there is still ongoing dialogue between different specialist infection societies (MSIS, ICM, EBJIS) regarding their scorings and recommended individual tests which are used for their PJI classifications, it is obvious that there are core group of tests which are recommended and agreed by most of these societies. It is therefore very important that these most widely and recommended tests are available locally and routinely used in the early diagnosis. These tests that are currently recommended by most of the infection societies include serum CRP and ESR, synovial fluid CC and PMN%, synovial fluid extended cultures, and should be done for every case when PJI is suspected. For some difficult to diagnose equivocal cases some additional investigations such histopathology, synovial Alpha-Defensin, next generation sequencing can also be done.

Once the correct diagnosis of PJI is established, it is crucial that—depending on the individual case, depending on the host, the bone defect, and the overall complexity of the case (see also Revision Knee Complexity Classification System)—the patient is getting the treatment at the right level of centre where the necessary expertise and conditions are available [18]. This is why regional revision networks are very important: they ensure that the patient is referred to the appropriate centre within the network, depending on the complexity of the individual case. For successful revision network development, complex planning and financing arrangements have to be put in place. Detailed and precise documentation of all cases include the treatment and the final outcome also play a significant role in constantly developing and improving the care that we provide for our patients.

The infected total knee replacement is a significant challenge for the surgeon and the whole multidisciplinary team, and it is a catastrophic, limb or life threatening complication for the patient. With early correct diagnosis, adequate classification of the particular case, and appropriate referral to the centre depending on the complexity of the revision, there is a good chance that most of these patients will receive successful treatment.

## References

[1] Abbas AM, Williams RL, Khan WS, Ghandour A, Morgan-Jones RL (2016). Tibial crest osteotomy in extensile knee exposure-a modified, low-energy, suture technique. J Arthroplasty.

[2] Berbari E, Mabry T, Tsaras G, Spangehl M, Erwin PJ, Murad MH, Steckelberg J, Osmon D (2010). Inflammatory blood laboratory levels as markers of prosthetic joint infection: a systematic review and meta-analysis. J Bone Joint Surg Am.

[3] Bozic KJ, Kurtz SM, Lau E, Ong K, Chiu V, Vail TP (2010). The epidemiology of revision total knee arthroplasty in the United States. Clin Orthop Rel Res.

[4] Chotanaphuti T, Courtney PM, Fram B, In den Kleef NJ, Kim TK, Kuo FC, Lustig S, Moojen DJ, Nijhof M, Oliashirazi A, Poolman R, Purtill JJ, Rapisarda A, Rivero-Boschert S, Veltman ES (2019). Hip and knee section, treatment, algorithm: proceedings of international consensus on orthopedic infections. J Arthroplasty..

[5] Citak M, Friedenstab J, Abdelaziz H, Suero EM, Zahar A, Salber J, Gehrke T (2019). Risk factors for failure after 1-stage exchange total knee arthroplasty in the management of periprosthetic joint infection. J Bone Joint Surg Am.

[6] Della Valle C, Parvizi J, Bauer TW, DiCesare PE, Evans RP, Segreti J (2011). American Academy of Orthopaedic surgeons clinical practice guideline on: the diagnosis of periprosthetic joint infections of the hip and knee. J Bone Joint Surg Am.

[7] Gehrke T, Alijanipour P, Parvizi J (2015). The management of an infected total knee arthroplasty. Bone Joint J..

[8] Gehrke T, Lausmann C, Citak M, Bonanzinga T, Frommelt L, Zahar A (2018). The accuracy of the alpha defensin lateral flow device for diagnosis of periprosthetic joint infection: comparison with a gold standard. J Bone Joint Surg Am.

[9] Goswami K, Parvizi J, Maxwell CP (2018). Current recommendations for the diagnosis of acute and chronic PJI for hip and knee-cell counts, alpha-defensin, leukocyte esterase next-generation sequencing. Curr Rev Musculoskelet Med.

[10] McNally M, Sousa R, Wouthuyzen-Bakker M, Chen AF, Soriano A, Vogely HC, Clauss M, Higuera CA, Trebše R (2021). The EBJIS definition of periprosthetic joint infection. Bone Joint J..

[11] McPherson EJ, Tontz W, Patzakis M, Woodsome C, Holtom P, Norris L, Shufelt C (1999). Outcome of infected total knee utilizing a staging system for prosthetic joint infection. Am J Orthop (Belle Mead NJ).

[12] Mühlhofer H, Renz N, Zahar A, Lüdemann M, Rudert M, Hube R, Frommelt L, Ascherl R, Perka C, von Eisenhart-Rothe R. (2020) (Diagnosis of periprosthetic joint infection : development of an evidence-based algorithm by the work group of implant-associated infection of the AE-(German Society for Arthroplasty)). Orthopade. DOI: 10.1007/s00132-020-03940-6. 10.1007/s00132-020-03940-6 PMC7990870 32666142

[13] Ohlmeier M, Filitarin S, Delgado G, Frings J, Abdelaziz H, Salber J, Frommelt L, Gehrke T, Citak M (2020). Improved treatment strategies can result in better outcomes following one-stage exchange surgery for MRSA periprosthetic joint infection. J Med Microbiol.

[14] Osmon DR, Berbari EF, Berendt AR, Lew D, Zimmerli W, Steckelberg JM (2013). Executive summary: diagnosis and management of prosthetic joint infection: clinical practice guidelines by the Infectious Diseases Society of America. Clin Infect Dis Off Publ Infect Dis Soc Am.

[15] Parvizi J, Gehrke T, Chen AF (2013). Proceedings of the international consensus on periprosthetic joint infection. Bone Joint J..

[16] Parvizi J, Tan TL, Goswami K, Higuera C, Della Valle C, Chen AF, Shohat N (2018). The 2018 definition of periprosthetic hip and knee infection: an evidence-based and validated criteria. J Arthroplasty.

[17] Parvizi J, Zmistowski B, Berbari EF, Bauer TW, Springer BD, Valle CJD (2011). New definition for periprosthetic joint infection: from the Workgroup of the Musculoskeletal Infection Society. Clin Orthop Rel Res.

[18] Phillips JRA, Al-Mouazzen L, Morgan-Jones R, Murray JR, Porteous AJ, Toms AD (2019). Revision knee complexity classification-RKCC: a common-sense guide for surgeons to support regional clinical networking in revision knee surgery. Knee Surg Sports Traumatol Arthrosc.

[19] Rossmann M, Minde T, Citak M, Gehrke T, Sandiford NA, Klatte TO, Abdelaziz H (2020). High rate of reinfection with new bacteria following one-stage exchange for enterococcalperiprosthetic infection of the knee: a single-center study. J Arthroplasty.

[20] Sandiford NA, McHale A, Citak M, Kendoff D (2020). What is the optimal duration of intravenous antibiotics following single-stage revision total hip arthroplasty for prosthetic joint infection? A systematic review. Hip Int.

[21] Shahi A, Parvizi J (2015). Prevention of periprosthetic joint infection. Arch Bone JtSurg.

[22] Wang C, Li R, Wang Q, Wang C (2018). Synovial fluid leukocyte esterase in the diagnosis of peri-prosthetic joint infection: a systematic review and meta-analysis. Surg Infect.

[23] Whiteside LA (1995). Exposure in difficult total knee arthroplasty using tibial tubercle osteotomy. Clin Orthop Relat Res.

[24] Zahar A, Kendoff DO, Klatte TO, Gehrke TA (2016). Can good infection control be obtained in one-stage exchange of the infected TKA to a rotating hinge design? 10-year results. ClinOrthopRelat Res.

[25] Zahar A, Lausmann C, Cavalheiro C, Dhamangaonkar AC, Bonanzinga T, Gehrke T, Citak M (2018). How reliable is the cell count analysis in the diagnosis of prosthetic joint infection?. J Arthroplasty.

